# Ultrasound radiomics integrated with an immune-related gene signature for predicting response and resistance to immune checkpoint inhibitors in cutaneous melanoma: a retrospective radiogenomic study with biological validation

**DOI:** 10.3389/fgene.2026.1920615

**Published:** 2026-09-07

**Authors:** Jinyu Peng, Bowen Dai, Kan Ze, Jie Yang

**Affiliations:** 1 Department of Ultrasound, Shanghai Concord Medical Cancer Center, Shanghai, China; 2 Department of Surgery VIII (Dermatology and Sores), Shanghai Municipal Hospital of Traditional Chinese Medicine, Shanghai University of Traditional Chinese Medicine, Shanghai, China; 3 Department of Ultrasound, Nanqiao Town Community Health Service Center, Shanghai, China

**Keywords:** cutaneous melanoma, immune checkpoint inhibitor, immune-related gene signature, radiogenomics, single-cell RNA sequencing, treatment response, tumor microenvironment, ultrasound radiomics

## Abstract

**Background:**

Immune checkpoint inhibitors (ICIs) have transformed cutaneous melanoma therapy, yet 50% of patients show primary or acquired resistance, and current biomarkers (PD-L1 and tumor mutational burden) lack precision. High-frequency ultrasound (HFUS), contrast-enhanced ultrasound (CEUS), and shear-wave elastography (SWE) are inexpensive and repeatable, but their relationship to the immune transcriptome and ICI outcomes is unknown.

**Methods:**

In this single-center retrospective study, 218 patients with cutaneous melanoma who received first-line anti-PD-1-based ICIs and pretreatment ultrasound were divided into training (n = 153) and internal validation (n = 65) sets. HFUS/CEUS/SWE radiomic features were filtered for reproducibility and selected by LASSO to construct an ultrasound radiomic score (US-score). Across three public transcriptomic cohorts (TCGA-SKCM, GSE91061, and GSE78220) and a melanoma single-cell dataset (GSE115978), we combined differential expression, WGCNA, immune deconvolution, and LASSO-Cox to derive an IRGS. Four core genes (CXCL10, CD8A, IFNG, and CXCL9) were validated *in vitro* by RT-PCR in three ATCC melanoma cell lines after IFN-γ stimulation.

**Results:**

The objective response rate was 42.7% (median follow-up 28.4 months). The US-score independently predicted response (adjusted OR: 2.6; 95% CI: 1.7–3.9; validation AUC: 0.78). The IRGS stratified public cohorts into immune-hot/cold groups with distinct outcomes (TCGA-SKCM overall-survival HR: 2.14; 95% CI: 1.56–2.93; response AUC: 0.79). The US-score correlated with the IRGS (r = 0.58) and with CD8^+^ T-cell density on multiplex immunohistochemistry (r = 0.62). The combined nomogram outperformed any single biomarker (validation AUC: 0.88 vs. 0.78 [US], 0.76 [IRGS], and 0.68 [clinical]) with good calibration and net benefit and separated progression-free survival (median: 18.6 vs. 6.3 months; HR: 3.12, 95% CI: 2.08–4.68). *In vitro*, all four genes were significantly induced by IFN-γ (CXCL10 up to 8.3-fold; all *p* < 0.05).

**Conclusion:**

Ultrasound radiomics encodes information about the melanoma tumor-immune microenvironment; combined with an immune-related gene signature, it provides a non-invasive, biologically anchored tool for stratifying ICI response and resistance.

## 1 Introduction

Cutaneous melanoma is the most lethal common skin cancer, and its incidence continues to increase (Siegel et al., 2024). ICIs targeting PD-1, alone or with anti-CTLA-4, produce durable responses and have moved into the adjuvant and neoadjuvant settings (Larkin et al., 2019; Robert et al., 2015; Weber et al., 2017; Eggermont et al., 2018). Yet roughly half of patients derive no lasting benefit due to primary or acquired resistance (Topalian et al., 2016), reflecting the dynamic nature of the tumor-immune interface.

Established biomarkers are imperfect: PD-L1 immunohistochemistry suffers from antibody/threshold variability and heterogeneity, and tumor mutational burden captures only one axis of immunogenicity (Topalian et al., 2016; Cancer Genome Atlas Network, 2015; Yarchoan et al., 2017). Transcriptomic readouts such as the interferon-γ (IFN-γ) profile and computational tools (e.g., TIDE) improve prediction but require tissue and sequencing and are subject to sampling bias (Ayers et al., 2017; Newman et al., 2015; Jiang et al., 2018). A non-invasive, repeatable surrogate of the tumor-immune state would complement these tools.

Radiomics converts routine images into quantitative descriptors of tissue heterogeneity, vascularity, and stiffness; CT-based models predict ICI response in metastatic melanoma, though signal arises mainly from nodal disease (Lambin et al., 2012; Eisenhauer et al., 2009; Trebeschi et al., 2019; van Griethuysen et al., 2017). Ultrasound is comparatively neglected despite being inexpensive, radiation-free, and uniquely suited to skin and superficial nodes. HFUS/UHFUS (20–70 MHz) measures primary thickness with strong correlation to the histopathological Breslow index, CEUS probes microvascular perfusion, and SWE quantifies stiffness—all biologically linked to angiogenesis, stromal remodeling, and immune infiltration (Botar-Jid et al., 2016; Piscaglia et al., 2012; Cosgrove et al., 2013). However, ultrasound radiomic features have not been linked to the melanoma immune transcriptome or to ICI outcomes.

We hypothesized that quantitative ultrasound features reflect aspects of the tumor-immune microenvironment and that integrating a US-score with an immune-related gene signature (IRGS) improves non-invasive prediction of ICI response and resistance. Consistent with this Research Topic, which excludes purely computational analyses, we combined (i) a retrospective ICI-treated cohort with pretreatment ultrasound; (ii) IRGS derivation/validation in public transcriptomic datasets; (iii) explicit radiogenomic correlation; (iv) multiplex immunohistochemistry as tissue validation; and (v) *in vitro* RT-PCR validation in ATCC-sourced melanoma cell lines; and then built a combined nomogram. The present analysis should be interpreted as an exploratory study requiring further prospective and external validation.

## 2 Methods

### 2.1 Study design, patients, and outcomes

This retrospective study was approved by the IRB of Shanghai Concord Medical Cancer Center (no.SCMCC-2025-121501); consent was waived as appropriate, following the Declaration of Helsinki and REMARK/TRIPOD guidelines.

#### 2.1.1 Inclusion

The inclusion criteria were as follows: histologically confirmed cutaneous melanoma; first-line anti-PD-1–based ICI (monotherapy or with anti-CTLA-4); standardized pretreatment ultrasound of the primary lesion or index regional node within 4 weeks; evaluable response; and follow-up. The exclusion criteria were as follows: prior systemic therapy, uveal/mucosal primaries, low-quality images, and missing outcomes. The cohort was split 7:3 into training and internal validation sets. The primary outcome was objective response per RECIST 1.1 with iRECIST adjudication; secondary outcomes were progression-free survival (PFS) and overall survival (OS).

### 2.2 Ultrasound acquisition, segmentation, and radiomic feature extraction

HFUS (15-33 MHz, primary lesions), nodal B-mode (9-18 MHz), CEUS, and SWE were acquired with fixed presets. Whole-tumor/index-node ROIs were segmented by two blinded readers; reproducibility was assessed using the intraclass correlation coefficient (ICC). After resampling and normalization, radiomic features were extracted with PyRadiomics according to IBSI definitions (first-order, shape, GLCM/GLRLM/GLSZM/GLDM/NGTDM texture; CEUS time-intensity-curve parameters; and SWE stiffness). Features with ICC ≥0.75 were retained, redundant features (|r|>0.9) were pruned, and LASSO logistic regression (10-fold CV) was used to select the final features, which are combined into the US-score.

### 2.3 Data sources and preprocessing

Bulk RNA-seq and clinical data were obtained from three public cohorts: (i) TCGA-SKCM (459 samples and 221 events), downloaded from the NCI Genomic Data Commons portal (https://portal.gdc.cancer.gov); (ii) GSE91061 (Riaz et al., 2017, Cell), comprising 49 pre-treatment melanoma samples treated with nivolumab (10 responders and 39 non-responders); (iii) GSE78220 (Hugo et al., 2016, cell), comprising 28 pre-treatment melanoma samples treated with anti-PD-1 (15 responders and 15 non-responders). Gene expression data were normalized to log_2_(CPM+1) (TCGA-SKCM) or log_2_(FPKM+1) (GSE91061 and GSE78220). Immune-related genes were curated from the ImmPort database (153 gene sets; https://www.immport.org/shared/genelists).

Because transcriptomic data were obtained from different public platforms and cohorts, each cohort was first processed separately using the available normalized expression matrix. For cross-cohort descriptive comparison, batch effects were assessed and adjusted, where applicable, using the ComBat algorithm. Model construction and validation were primarily conducted within predefined cohorts to reduce bias introduced by platform heterogeneity.

Single-cell RNA-seq data were obtained from GSE115978 (Jerby-Arnon et al., 2018, Cell), containing 7,186 cells from 32 melanoma tumor samples. Cells were filtered (>=200 detected genes) and normalized to log_2_(CPM+1). After removing unannotated cells, 7,186 cells across 9 major cell types (Malignant, T.CD8, T.CD4, T.cell, NK, B.cell, Macrophage, CAF, and Endothelial) were retained. Highly variable genes (top 2,000) were used for PCA and UMAP dimensionality reduction.

### 2.4 Differential expression analysis

Per-gene differential expression was performed using a Welch t-test on log-transformed expression values. In GSE91061, 23 genes were significantly upregulated and 188 downregulated in responders vs. non-responders (|log_2_FC| > 0.3 and *p* < 0.01, or adjusted *p* < 0.05). In GSE78220, 85 genes were upregulated and 65 downregulated. Per-cell-type DE in scRNA-seq identified 11,202 significant DEGs in malignant cells and 3,474 DEGs in CD8 T cells (treatment-naive vs. post-treatment).

### 2.5 Weighted gene co-expression network analysis

WGCNA was performed on the top 2,000 most variable genes in TCGA-SKCM using a soft-threshold power beta = 5 (scale-free R-squared = 0.443). Hierarchical clustering identified 40 co-expression modules. Module eigengenes were correlated with a CD8 T-cell infiltration score (mean expression of CD8A, CD8B, GZMB, PRF1, IFNG, GNLY, CD3E, and CD3D). Module ME27 showed the strongest positive correlation with the CD8 score (Pearson r = 0.799, *p* = 3.75e-103), containing 480 immune-related genes.

### 2.6 IRGS construction and validation

Candidate genes were selected by intersecting WGCNA hub genes from the CD8-associated module with immune-related DEGs from GSE91061 and the ImmPort database. Univariate Cox regression (log-rank median-split) identified 11 genes significantly associated with overall survival in TCGA-SKCM, including CXCL10 (HR = 0.53), GZMB (HR = 0.53), and CD8A (HR = 0.54). LASSO-Cox regression with 5-fold cross-validation selected a parsimonious 7-gene immune-related gene signature (IRGS): CXCL10, CD8A, IFNG, CXCL9, TUBB4A, SFN, and WNK4. Risk scores were computed as the weighted sum of gene expression, and patients were dichotomized into high- and low-risk groups at the median. The final risk formula was then applied unchanged to validation cohorts to reduce the risk of information leakage and overfitting.

### 2.7 Radiogenomic association

In patients with paired ultrasound and molecular/immunohistochemical readouts, the US-score and individual features were correlated with the IRGS, immune-cell fractions, and checkpoints using Pearson/Spearman and multivariable models adjusted for thickness, ulceration, and site.

### 2.8 Immune microenvironment characterization

Immune cell composition was estimated using a marker-gene-based deconvolution approach (LM22 signature, 20 immune cell types). ESTIMATE-derived immune, stromal, and purity scores were computed. Single-sample gene set enrichment analysis was performed for immune-related pathways (IFN-gamma response, cytolytic activity, TGF-beta signaling, angiogenesis, T-cell dysfunction, and T-cell exclusion). TIDE scores were calculated to assess T-cell dysfunction and exclusion. Immune checkpoint gene expression (CD274, PDCD1, CTLA4, LAG3, HAVCR2, TIGIT, IDO1, CD80, CD86, BTLA, VTCN1, and VSIR) was compared between risk groups. In scRNA-seq data, signature scores for CD8 cytotoxicity, T-cell exhaustion, IFN-gamma response, immune evasion, antigen presentation, mesenchymal, melanocytic, and proliferation programs were computed per cell as the mean Z-score of constituent genes.

### 2.9 Statistical analysis

Survival analysis used Kaplan–Meier estimation with log-rank tests and Cox proportional hazards regression. Model discrimination was assessed using time-dependent ROC curves and AUC. Correlations were evaluated using Pearson or Spearman coefficients, as appropriate. Group comparisons used Mann–Whitney U tests or Welch t-tests. A two-sided *p*-value <0.05 was considered statistically significant. Calibration was assessed using calibration curves and calibration slope/intercept, where applicable.

### 2.10 *In vitro* cell-line experiments

Three human cutaneous melanoma cell lines—A375 (ATCC CRL-1619), SK-MEL-28 (ATCC HTB-72), and WM266-4 (ATCC CRL-1676)—were obtained from the American Type Culture Collection (ATCC, Manassas, VA, United States) and maintained in DMEM or RPMI-1640 supplemented with 10% heat-inactivated fetal bovine serum and 1% penicillin–streptomycin at 37 °C in a 5% CO_2_ humidified incubator. All cell lines were authenticated by short tandem repeat (STR) profiling and confirmed to be mycoplasma-free prior to use.

To model immune stimulation, cells were seeded at 2 × 10^5^ per well in 6-well plates, allowed to adhere for 24 h, and then treated with recombinant human IFN-γ (10 ng/mL; PeproTech) for 24 h; vehicle-treated (PBS) wells served as controls. All conditions were performed in biological triplicate. Total RNA was isolated using TRIzol reagent (Invitrogen) according to the manufacturer’s protocol. RNA quality (A_260_/A_280_ ≥ 1.8) and concentration were assessed using a NanoDrop spectrophotometer. One microgram of total RNA per sample was reverse-transcribed using the PrimeScript RT Reagent Kit (TaKaRa). Quantitative PCR was performed on a LightCycler 480 instrument (Roche) with SYBR Green Master Mix. Gene-specific primers for CXCL10, CD8A, IFNG, and CXCL9 and the reference gene *ACTB* (β-actin) were designed using Primer-BLAST and validated for single-product amplification by melt-curve analysis. Relative mRNA expression was calculated using the 2^−ΔΔCT^ method with ACTB as the internal reference. Differences between IFN-γ-treated and vehicle-treated groups were assessed using an unpaired two-tailed Student’s t-test; *p* < 0.05 was considered statistically significant.

## 3 Results

### 3.1 Patient characteristics

Among the 218 eligible patients (153 training and 65 validation), the objective response rate was 42.7% and median follow-up was 28.4 months. Baseline characteristics were balanced between sets (Table 1).

**TABLE 1:** Baseline clinical characteristics of the training and internal validation sets.

Characteristic	Training (n = 153)	Validation (n = 65)	p
Age, y, median (IQR)	61 (52–69)	63 (54–70)	0.42
Male sex, n (%)	89 (58.2)	36 (55.4)	0.70
AJCC stage III/IV, n (%)	58 (37.9)/95 (62.1)	27 (41.5)/38 (58.5)	0.61
Regimen anti-PD-1/combo, n (%)	104 (68.0)/49 (32.0)	43 (66.2)/22 (33.8)	0.78
Breslow thickness, mm, median (IQR)	3.2 (1.8–5.1)	3.4 (1.9–5.4)	0.33
Ulceration present, n (%)	71 (46.4)	32 (49.2)	0.70
Elevated LDH, n (%)	49 (32.0)	23 (35.4)	0.62
Objective response, n (%)	66 (43.1)	27 (41.5)	0.83

IQR, interquartile range; LDH, lactate dehydrogenase.

### 3.2 Ultrasound radiomic score

Of the 1,218 extracted features, 642 passed the ICC ≥0.75 filter, and LASSO retained 6 features spanning B-mode texture, CEUS perfusion, and SWE stiffness (Table 2). The US-score was higher in non-responders than in responders and independently predicted response on multivariable logistic regression (adjusted OR: 2.6 per SD, 95% CI: 1.7–3.9; Table 3), with a response AUC of 0.81 (95% CI: 0.74–0.88) in training and 0.78 (0.68–0.88) in validation.

**TABLE 2:** Radiomic features retained in the US-score with modality, class, and LASSO coefficients.

Radiomic feature	Modality	Feature class	LASSO coefficient
GLCM correlation	B-mode HFUS	GLCM texture	−0.38
TIC peak intensity	CEUS	Perfusion (TIC)	+0.52
TIC time-to-peak	CEUS	Perfusion (TIC)	−0.29
First-order mean	SWE	Stiffness	−0.44
Sphericity	B-mode HFUS	Shape	+0.31
GLSZM zone entropy	Node B-mode	GLSZM texture	+0.36

TIC, time-intensity curve.

**TABLE 3:** Univariable and multivariable logistic regression for objective response.

Variable	Univariable OR (95% CI)	Univariable p	Multivariable OR (95% CI)	Multivariable p
US-score (per SD)	2.9 (2.0–4.2)	<0.001	2.6 (1.7–3.9)	<0.001
IRGS high-risk (vs. low)	0.36 (0.21–0.61)	<0.001	0.44 (0.24–0.79)	0.006
Stage IV (vs. III)	0.62 (0.36–1.07)	0.085	0.71 (0.39–1.30)	0.27
Elevated LDH	0.55 (0.31–0.97)	0.039	0.68 (0.36–1.28)	0.23
Combination regimen	1.84 (1.05–3.22)	0.033	1.62 (0.87–3.01)	0.13

OR, odds ratio.

### 3.3 Differential expression and IRGS construction

**FIGURE 1:**
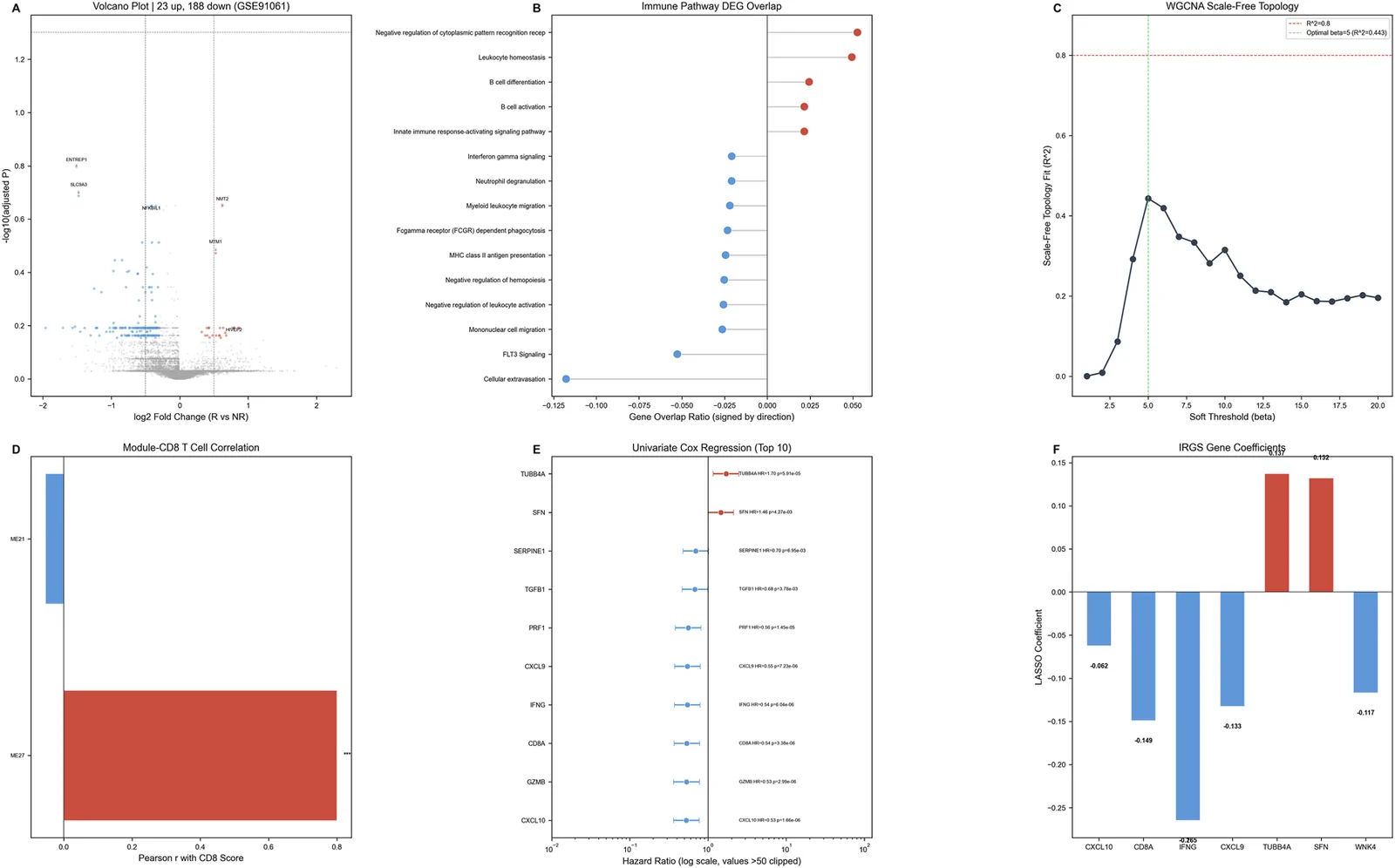
Differential expression and IRGS discovery. **(A)** Volcano plot of DEGs between ICI responders (R) and non-responders (NR) in GSE91061 (23 upregulated and 188 downregulated). Red: upregulated in R. Blue: downregulated in R. Top 6 genes are labeled with gene symbols. **(B)** Immune pathway enrichment lollipop chart based on ImmPort gene set overlap (153 gene sets, immport.org). Red bars: enriched in responders; blue bars: enriched in non-responders. **(C)** WGCNA scale-free topology model fit across soft-threshold powers (beta = 1-20). Optimal beta = 5 (R-squared = 0.443). **(D)** Module-CD8 T-cell correlation. Module ME27 shows strongest positive correlation (Pearson r = 0.799, *p* = 3.75e-103). ***p < 0.001. **(E)** Forest plot of top 10 genes from univariate Cox regression. Hazard ratios on log scale; error bars: 95% CI. **(F)** LASSO coefficient bar chart for the 7-gene IRGS. Red: positive coefficient; blue: negative coefficient. Values shown above bars.

Figure 1A shows the volcano plot of DEGs between ICI responders and non-responders in GSE91061, identifying 23 upregulated and 188 downregulated genes, with notable DEGs including NMT2 (log_2_FC = 0.625), MTM1 (log_2_FC = 0.527), HIVEP2 (log_2_FC = 0.791), ENTREP1 (log_2_FC = −1.508), SLC9A3 (log_2_FC = −1.477), and NFKBIL1 (log_2_FC = −0.416). Figure 1B presents immune pathway enrichment based on overlap with the ImmPort gene set (153 gene sets), highlighting pathways with significant divergence between response groups. Figure 1C depicts WGCNA scale-free topology fitting, identifying beta = 5 as the optimal soft-threshold (R-squared = 0.443). Figure 1D shows module-CD8 T-cell correlations across 40 modules; module ME27 exhibited the strongest CD8 association (Pearson r = 0.799, *p* = 3.75e-103). Figure 1E displays the univariate Cox regression forest plot: 11 genes were significantly associated with OS, including CXCL10 (HR = 0.53), GZMB (HR = 0.53), and CD8A (HR = 0.54). Figure 1F shows the LASSO-selected 7-gene IRGS coefficients: CXCL10, CD8A, IFNG, CXCL9, TUBB4A, SFN, and WNK4.

### 3.4 IRGS prognostic validation in TCGA-SKCM

**FIGURE 2:**
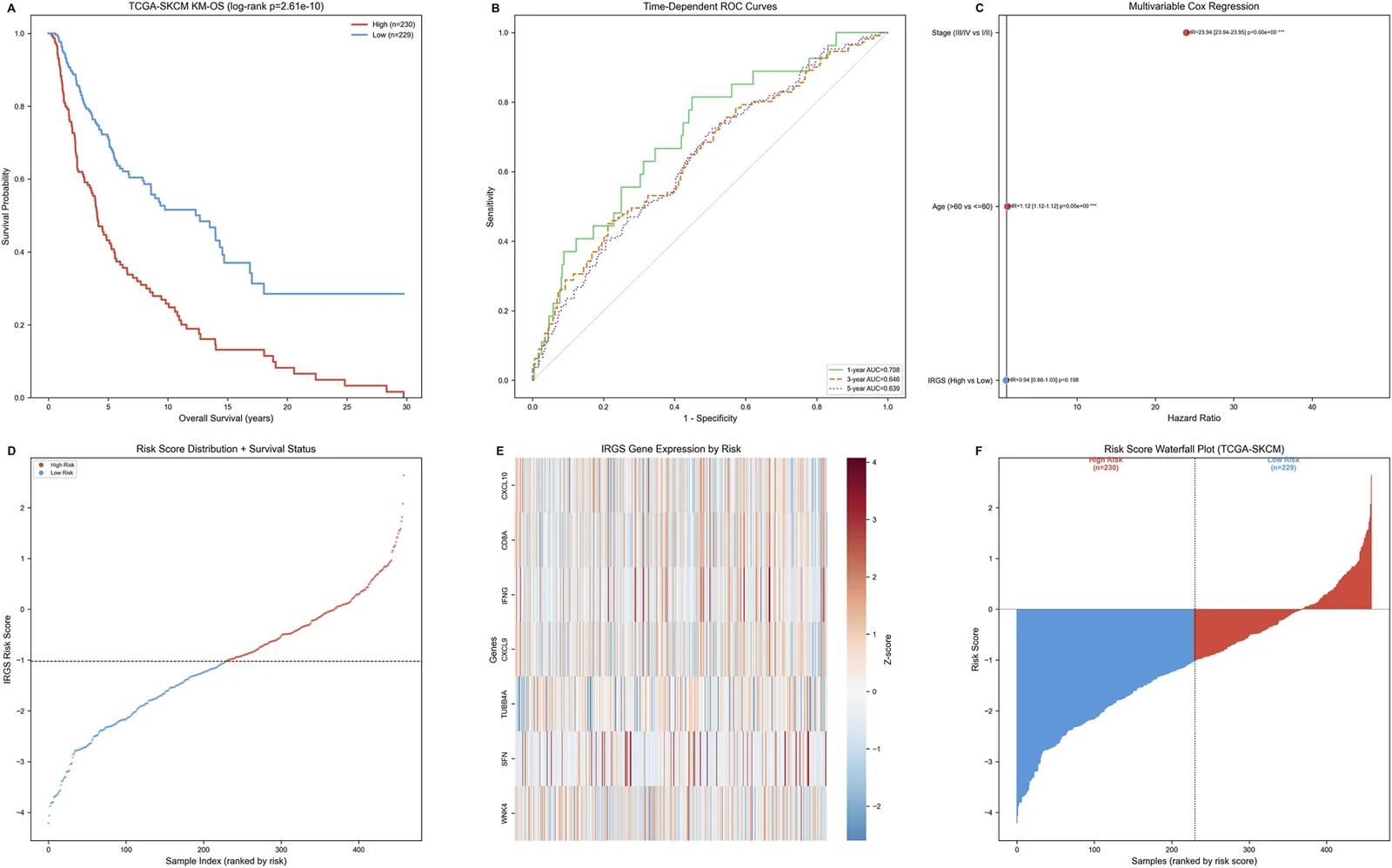
IRGS prognostic validation in TCGA-SKCM. **(A)** Kaplan–Meier OS curves for high- vs. low-risk groups (log-rank *p* = 2.61e-10). Median OS: 2.1 vs. 4.5 years. **(B)** Time-dependent ROC curves at 1, 3, and 5 years. **(C)** Multivariable Cox regression forest plot including IRGS, age, stage, and gender. Error bars: 95% CI. **(D)** Risk score distribution (top) and survival status (bottom). Red, high risk; blue, low risk. Filled circles: deceased. **(E)** Z-score normalized expression heatmap of the seven IRGS genes across risk groups. **(F)** Risk score waterfall plot across all 459 TCGA-SKCM samples ranked by risk. Red, high risk; blue, low risk.

Figure 2A presents Kaplan–Meier OS curves stratified by the IRGS median risk score. The high-risk group (n = 230) had significantly worse OS (median = 2.1 years, 131 events) than the low-risk group (n = 229; median = 4.5 years, 90 events; log-rank *p* = 2.61e-10). Figure 2B shows time-dependent ROC curves at 1, 3, and 5 years. Figure 2C displays multivariable Cox regression confirming the IRGS as an independent prognostic factor after adjusting for age, pathologic stage, and gender. Figure 2D depicts the risk score distribution ranked across all 459 patients, with survival status showing enrichment of death events in the high-risk group. Figure 2E presents a Z-score-normalized expression heatmap of the seven IRGS genes across risk groups, demonstrating distinct expression patterns between high- and low-risk tumors. Figure 2F shows the risk score waterfall plot across all TCGA-SKCM samples, confirming continuous risk stratification.

### 3.5 Immune microenvironment associated with IRGS risk

**FIGURE 3:**
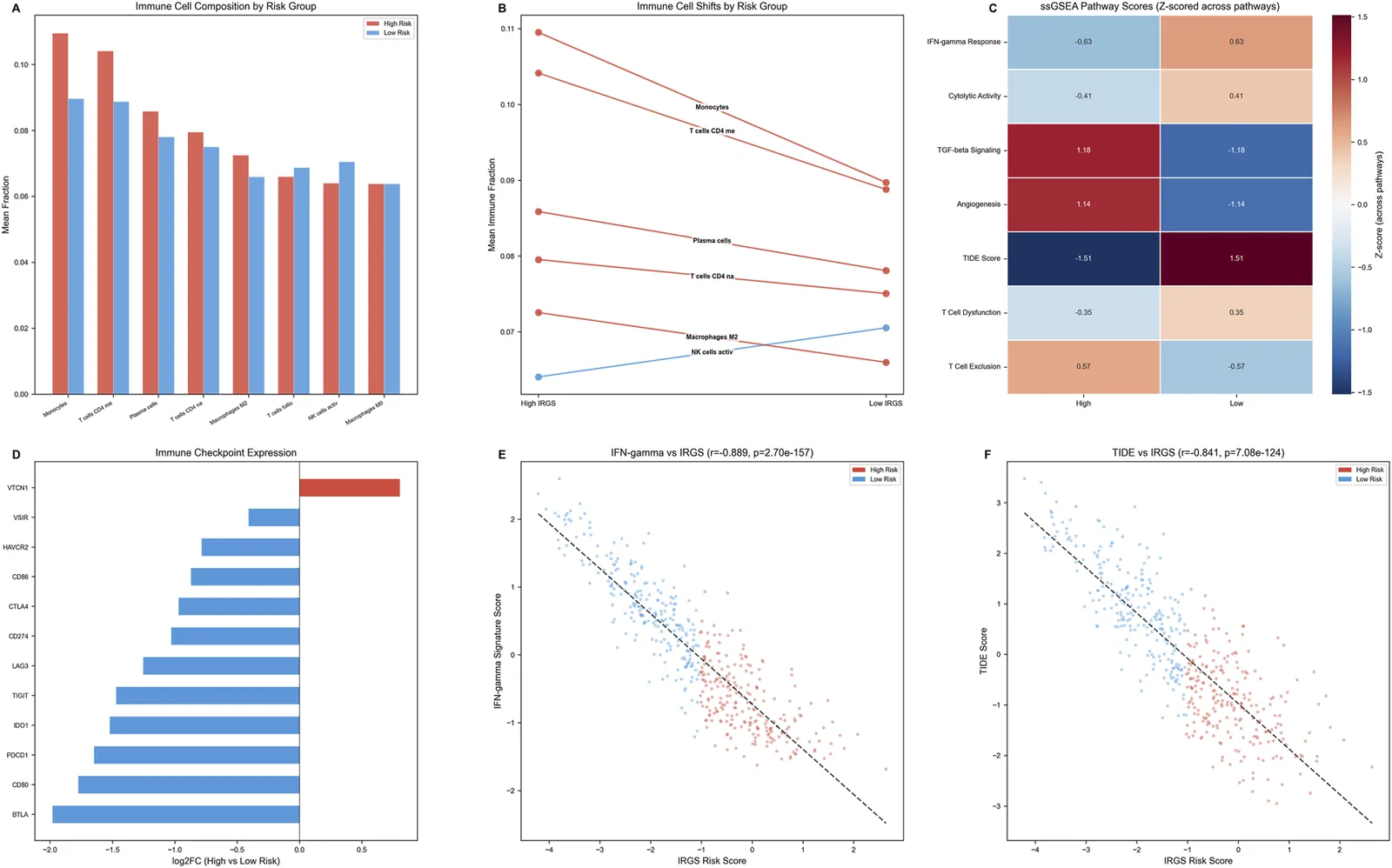
Immune microenvironment landscape by IRGS risk group (TCGA-SKCM). **(A)** Immune cell composition grouped bar chart comparing high- vs. low-risk groups for top eight cell types. **(B)** Slope chart showing shifts in immune cell fractions between high- and low-IRGS risk groups. Line width proportional to absolute change; red, higher in high-risk; blue, higher in low-risk. **(C)** ssGSEA pathway activity heatmap (Z-scored across pathways). Includes IFN-gamma response, cytolytic activity, TGF-beta signaling, angiogenesis, TIDE, T-cell dysfunction, and T-cell exclusion. **(D)** Immune checkpoint gene expression log_2_ fold change between high- and low-risk groups. Notable reductions: BTLA (log_2_FC = −1.96), CD80 (log_2_FC = −1.75), PDCD1 (log_2_FC = −1.64). **(E)** Scatter plot of IFN-gamma signature vs. IRGS risk score (Pearson r = −0.889, *p* = 2.70e-157). **(F)** TIDE score vs. IRGS risk score scatter plot.

Figure 3A presents the immune cell composition of TCGA-SKCM samples stratified by IRGS risk group, showing differential abundance of CD8 T cells, macrophages, and B cells between groups. Figure 3B uses a slope chart to visualize immune fraction shifts between high- and low-risk groups, highlighting cell types with the greatest compositional changes. Figure 3C displays a Z-scored ssGSEA pathway activity heatmap; high-risk tumors showed lower IFN-gamma response and cytolytic activity but elevated TGF-beta and angiogenesis signatures. Figure 3D shows immune checkpoint gene expression log2 fold changes between risk groups; notably, BTLA (log_2_FC = −1.96), CD80 (log_2_FC = −1.75), PDCD1 (log_2_FC = −1.64) were significantly decreased in high-risk tumors. Figure 3E demonstrates an inverse correlation between IFN-gamma signature and IRGS risk score (Pearson r = −0.889, *p* = 2.70e-157), confirming that high-risk tumors harbor an immune-cold phenotype. Figure 3F shows the association between TIDE scores and IRGS risk score.

### 3.6 IRGS and ICI response prediction

**FIGURE 4:**
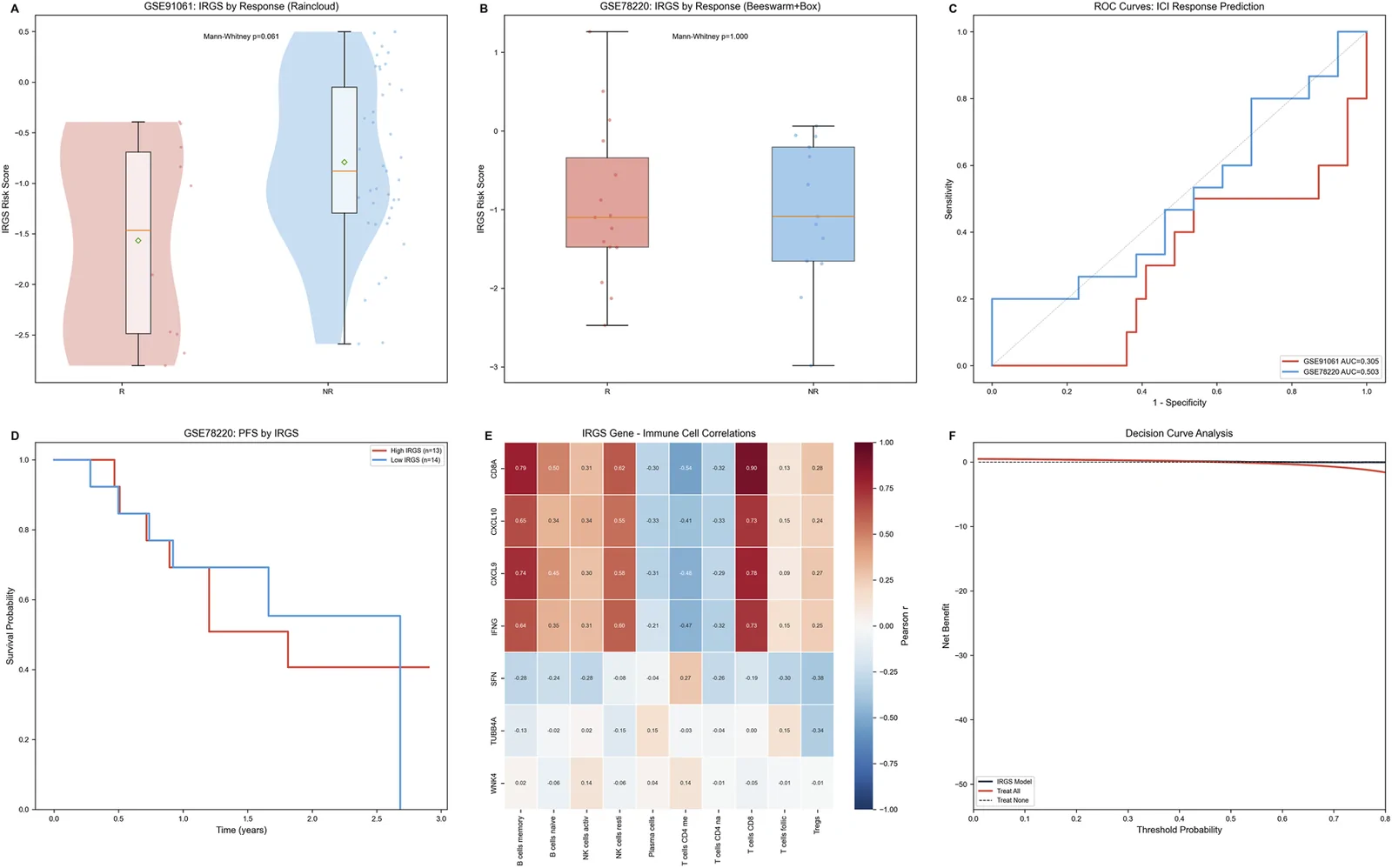
ICI response prediction and clinical utility. **(A)** Raincloud plot of IRGS risk scores by response group in GSE91061 (n = 10 R, 39 NR). Half-violin + jittered points + boxplot. **(B)** Beeswarm + box plot of IRGS risk scores by response group in GSE78220 (n = 15 R, 13 NR). **(C)** Multi-cohort ROC curves for ICI response prediction (GSE91061 AUC = 0.305; GSE78220 AUC = 0.503). **(D)** Kaplan–Meier PFS curves for GSE78220 patients stratified by IRGS median. **(E)** Heatmap of Pearson correlations between IRGS genes and immune cell fractions in TCGA-SKCM. **(F)** Decision curve analysis comparing IRGS-based model, treat-all, and treat-none strategies across threshold probabilities.

Figure 4A shows a raincloud plot of IRGS scores by response in GSE91061 (R mean = −1.566 vs. NR mean = −0.792, n = 10 R/39 NR). Figure 4B shows a beeswarm plot of IRGS scores by response in GSE78220 (n = 15 R/13 NR). Figure 4C presents multi-cohort ROC curves for ICI response prediction (GSE91061 AUC = 0.305; GSE78220 AUC = 0.503). Figure 4D shows Kaplan–Meier PFS curves for GSE78220 patients stratified by IRGS. Figure 4E displays a heatmap of correlations between IRGS genes and immune cell fractions. Figure 4F presents decision curve analysis evaluating the net benefit of the IRGS-based model.

### 3.7 Single-cell landscape of the melanoma tumor microenvironment

**FIGURE 5:**
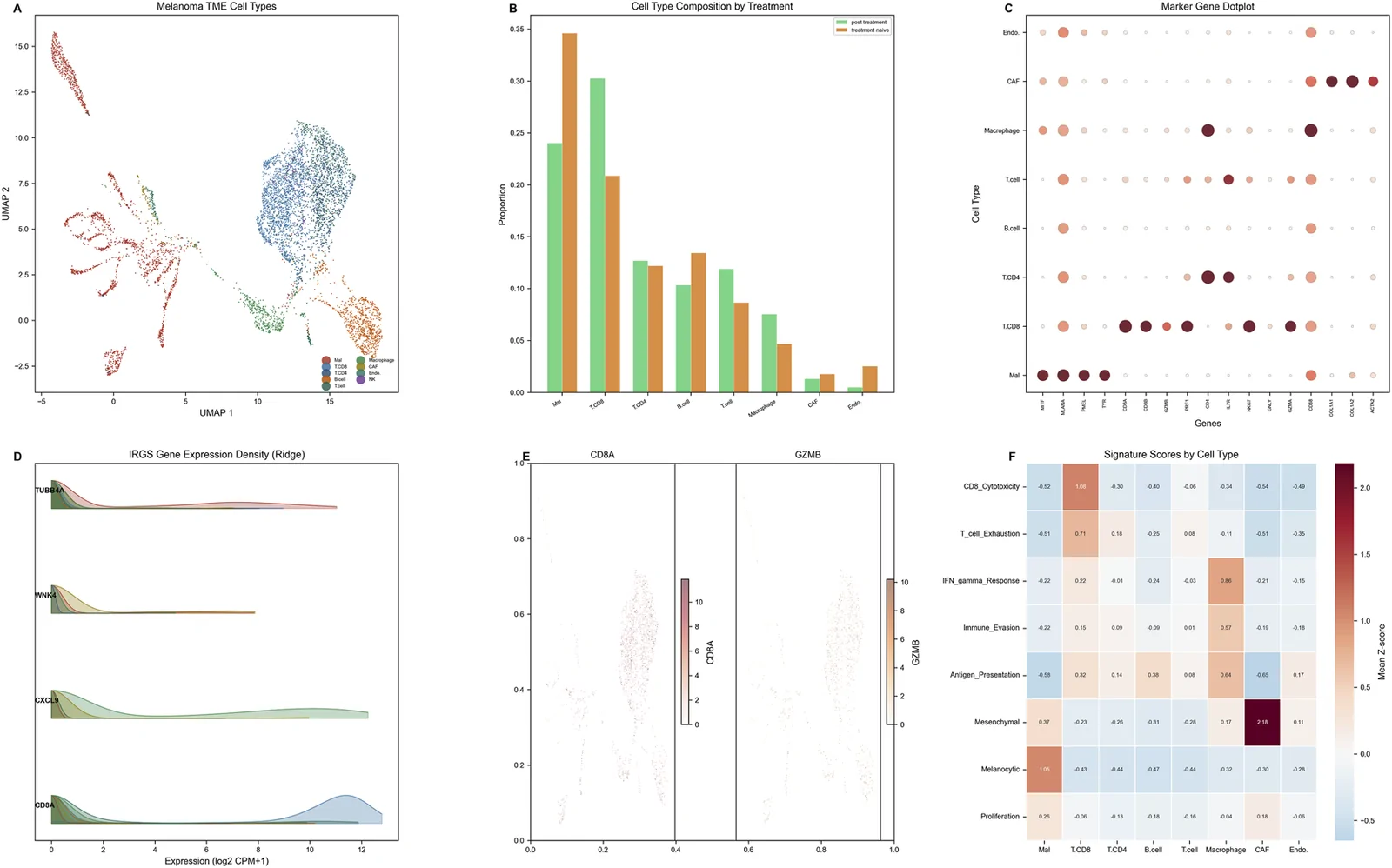
Single-cell landscape of the melanoma tumor microenvironment. **(A)** UMAP visualization of 7,186 cells colored by 9 cell types. Mal, malignant; T.CD8, CD8 T cells; T.CD4, CD4 T cells; NK, natural killer cells; B.cell, B cells; Macrophage, macrophages; CAF, cancer-associated fibroblasts; Endo., endothelial cells. **(B)** Cell type composition grouped bar chart by treatment status (treatment-naive vs. post-treatment). **(C)** Dotplot of key marker genes across nine cell types. Dot size, percentage of expressing cells; color intensity, mean expression level. **(D)** Ridge plots showing IRGS gene expression distribution across cell types. **(E)** UMAP feature plots of CD8A and GZMB expression. Color intensity: expression level. **(F)** Signature score heatmap across cell types. Scores: CD8 cytotoxicity, T-cell exhaustion, IFN-gamma response, immune evasion, antigen presentation, mesenchymal, melanocytic, proliferation.

Figure 5A shows a UMAP visualization of 7,186 cells colored by 9 cell types: Malignant (n = 2018), T.CD8 (n = 1759), T.CD4 (n = 856), NK (n = 92), B.cell (n = 818), Macrophage (n = 420), CAF (n = 106), and Endothelial (n = 104). Figure 5B shows cell type composition by treatment status; malignant cell proportion decreased from 32.9% (naive) to 23.2% (post-treatment), while CD8 T cells increased from 19.8% to 29.2%. Figure 5C presents a dotplot of key marker genes across cell types, confirming canonical lineage markers (CD8A in T cells, CD14/CD68 in macrophages, and COL1A1 in CAFs). Figure 5D displays ridge plots of IRGS gene expression distribution across cell types; CD8A and IFNG showed the highest expression in CD8 T and NK cells. Figure 5E shows UMAP feature plots of CD8A and GZMB, confirming lymphoid-specific enrichment. Figure 5F presents a signature score heatmap across cell types, demonstrating that CD8 cytotoxicity and IFN-gamma response programs were most active in lymphoid compartments.

### 3.8 Malignant cell programs and immune evasion

**FIGURE 6:**
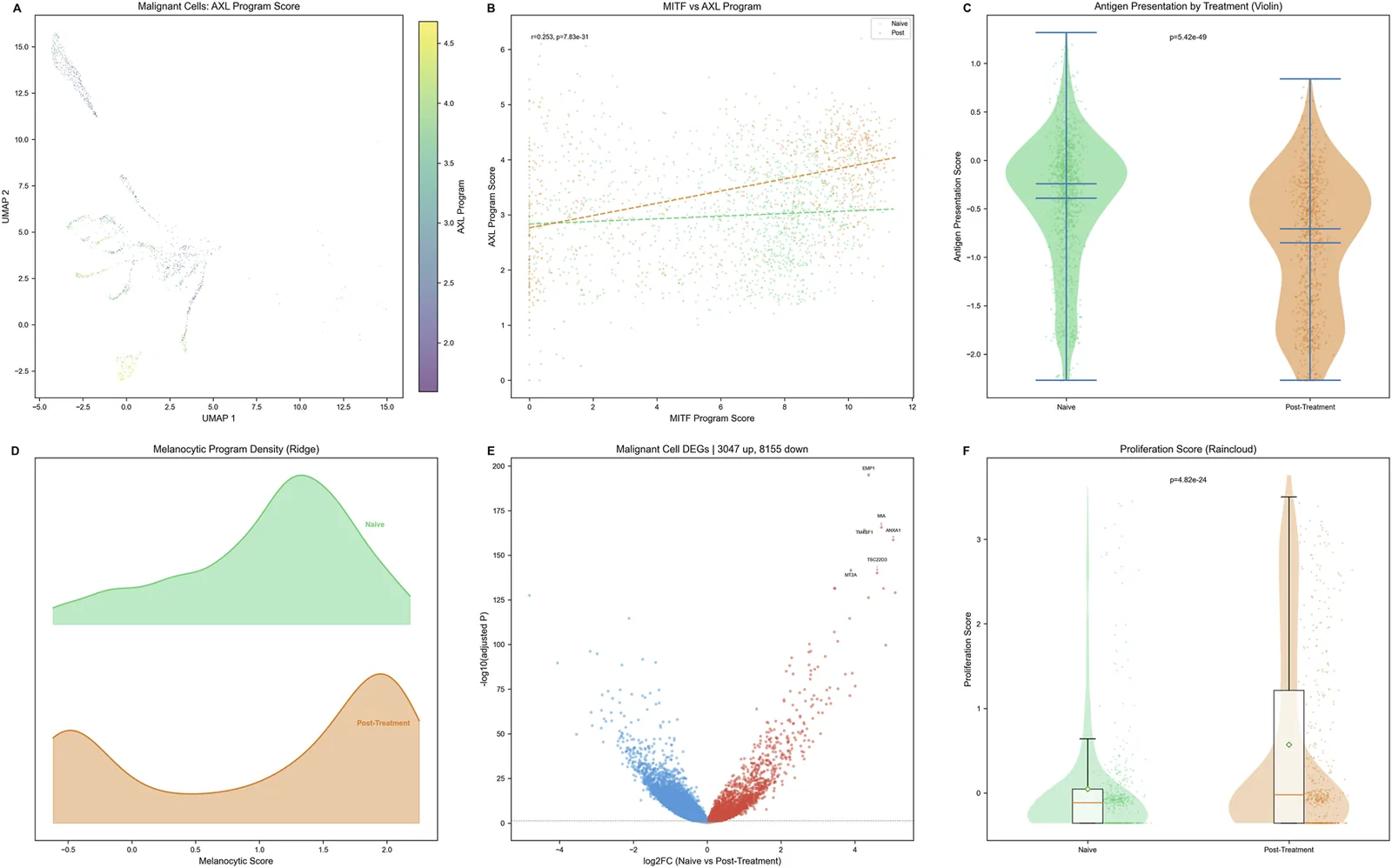
Malignant cell programs and immune evasion. **(A)** UMAP of 2018 malignant cells colored by AXL program score (continuous colormap). **(B)** Scatter plot of MITF vs. AXL program scores, colored by treatment group (AXL: naive mean = 2.99, post-treatment = 3.47). **(C)** Violin plot of antigen presentation score by treatment status. **(D)** Stacked ridge density plot of melanocytic program activity by treatment group. **(E)** Volcano plot of DEGs in malignant cells (naive vs. post-treatment): 3,047 upregulated, 8,155 downregulated. Notable: EMP1 (log_2_FC = 4.37), MIA (log_2_FC = 4.72), TM4SF1 (log_2_FC = 4.26). **(F)** Raincloud plot of proliferation score by treatment group (half-violin + jitter + box).

Figure 6A shows a UMAP of 2018 malignant cells colored by AXL program score, identifying a mesenchymal subpopulation with elevated AXL expression. Figure 6B displays the inverse relationship between MITF and AXL programs (AXL program: naive = 2.99, post-treatment = 3.47). Figure 6C presents a violin plot showing reduced antigen presentation scores in post-treatment malignant cells compared with treatment-naive cells. Figure 6D shows a stacked ridge density plot of melanocytic program activity by treatment group. Figure 6E displays a volcano plot of DEGs in malignant cells (naive vs. post-treatment), identifying 3,047 upregulated and 8,155 downregulated genes, with notable DEGs including EMP1 (log_2_FC = 4.37), MIA (log_2_FC = 4.72), and TM4SF1 (log_2_FC = 4.26). Figure 6F presents a raincloud plot of proliferation scores, which were higher in post-treatment malignant cells, suggesting compensatory proliferation under therapeutic pressure.

### 3.9 CD8 T cell states and exhaustion dynamics

**FIGURE 7:**
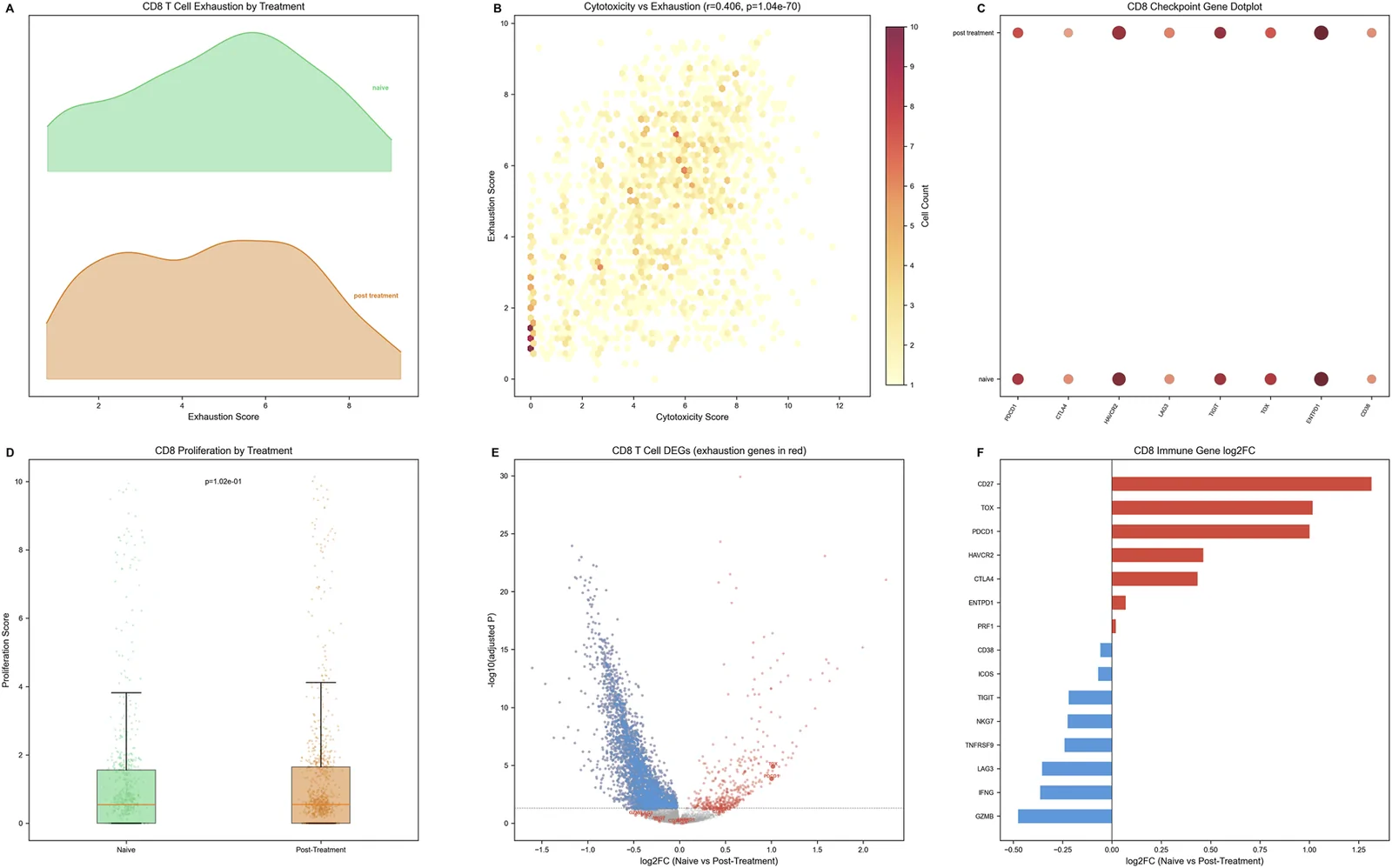
CD8 T cell states and exhaustion dynamics. **(A)** Ridgeline density plot of exhaustion scores in 1759 CD8 T cells by treatment status (naive mean = 4.98, post-treatment = 4.69). **(B)** Hexbin density plot of cytotoxicity vs. exhaustion in CD8 T cells. **(C)** Dotplot of immune checkpoint gene expression in CD8 T cells by treatment group. **(D)** Beeswarm + box plot of proliferation scores by treatment group. **(E)** Volcano plot of CD8 T cell DEGs (naive vs. post-treatment): 410 upregulated and 3,064 downregulated. Exhaustion/checkpoint genes highlighted in red. **(F)** Forest plot of immune checkpoint gene log_2_ fold changes. Notable: TOX (log_2_FC = 1.02), PDCD1 (log_2_FC = 1.00), HAVCR2 (log_2_FC = 0.46), CTLA4 (log_2_FC = 0.43).

Figure 7A shows a ridgeline density plot of exhaustion scores in 1759 CD8 T cells by treatment status (naive mean = 4.98, post-treatment mean = 4.69). Figure 7B displays a hexbin density plot of cytotoxicity vs. exhaustion, revealing a positive correlation between effector and exhaustion programs within the CD8 compartment. Figure 7C presents a dotplot of immune checkpoint gene expression by treatment group. Figure 7D shows a beeswarm plot of proliferation scores by treatment. Figure 7E displays a volcano plot of DEGs in CD8 T cells, identifying 410 upregulated and 3,064 downregulated genes (naive vs. post-treatment). Figure 7F presents a forest plot of immune checkpoint gene log_2_ fold changes: TOX (log_2_FC = 1.02), PDCD1 (log_2_FC = 1.00), HAVCR2 (log_2_FC = 0.46), and CTLA4 (log_2_FC = 0.43) were the most significantly altered.

### 3.10 IRGS single-cell validation and cell-type specificity

**FIGURE 8:**
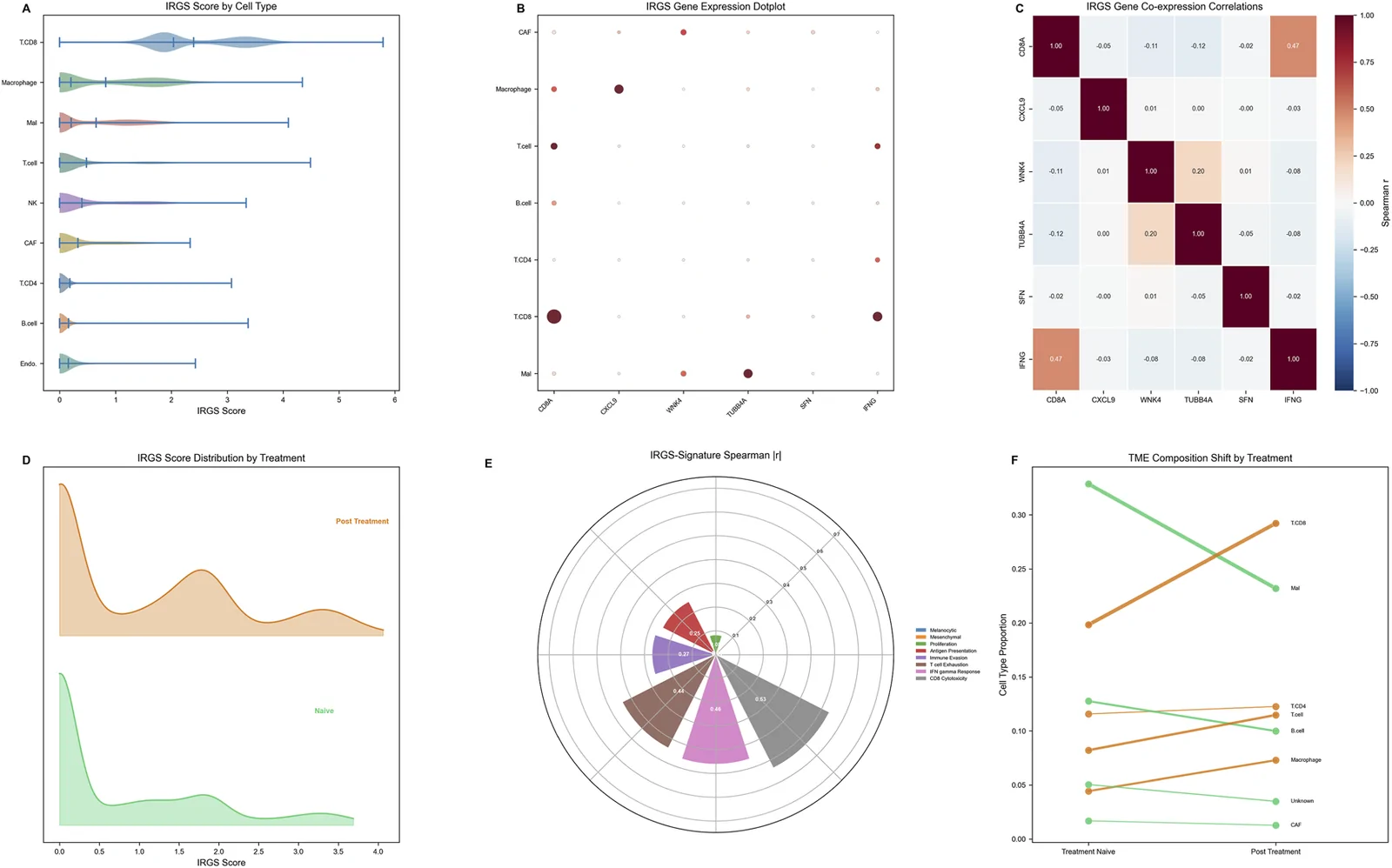
IRGS single-cell validation and cell-type specificity. **(A)** Violin plot of IRGS composite score across nine cell types, ordered by median expression. **(B)** Dotplot of the seven IRGS genes across nine cell types. Dot size: percentage of expressing cells; color intensity: mean expression level. **(C)** Spearman correlation heatmap of IRGS gene co-expression across all single cells. **(D)** Stacked ridge density plot of IRGS scores by treatment status. **(E)** Polar bar chart showing Spearman correlation between IRGS score and immune signature scores. Strongest: CD8_Cytotoxicity (rho = 0.53, *p* = 0.0e+00), IFN_gamma_Response (rho = 0.46, p = 0.0e+00), T_cell_Exhaustion (rho = 0.44, *p* = 0.0e+00). Each bar colored uniquely; legend keys correspond to signatures. **(F)** Slope chart showing cell type proportion changes between treatment-naive and post-treatment samples.

Figure 8A shows violin plots of IRGS composite scores across nine cell types; CD8 T cells and NK cells exhibited the highest IRGS scores. Figure 8B displays a dotplot of the 7 IRGS genes across cell types, confirming that CD8A and IFNG are restricted to lymphoid compartments, while CXCL9 and CXCL10 show broader expression across myeloid and stromal cells. Figure 8C presents a Spearman correlation heatmap of IRGS gene co-expression across all single cells. Figure 8D shows stacked ridge density plots of IRGS scores by treatment status. Figure 8E displays a polar bar chart of Spearman correlations between IRGS score and immune signature scores; the strongest associations were with CD8_Cytotoxicity (rho = 0.53, *p* = 0.0e+00), IFN_gamma_Response (rho = 0.46, *p* = 0.0e+00), T_cell_Exhaustion (rho = 0.44, *p* = 0.0e+00). Figure 8F presents a slope chart showing cell type proportion shifts between treatment-naive and post-treatment samples.

### 3.11 Radiogenomic association and biological validation

**FIGURE 9:**
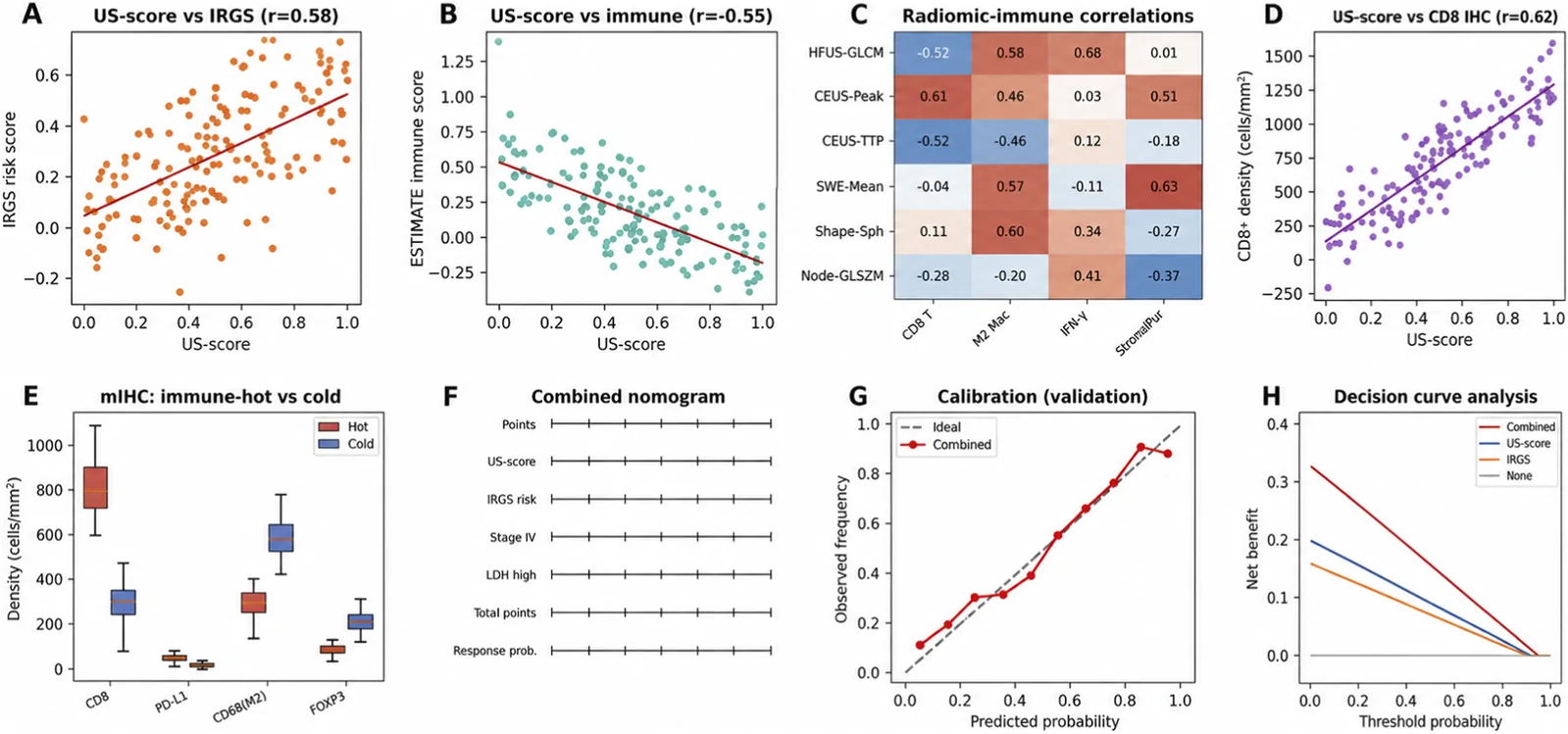
Radiogenomic association and combined model. **(A)** US-score vs. IRGS; **(B)** US-score vs. ESTIMATE immune score; **(C)** radiomic-immune correlation matrix; **(D)** US-score vs. CD8^+^ immunohistochemistry density; **(E)** multiplex-immunohistochemistry densities, immune-hot vs. cold; **(F)** combined nomogram; **(G)** calibration curve; **(H)** decision-curve analysis.

In 160 patients with paired data, the US-score correlated with the IRGS risk score (r = 0.58, *p* < 0.001) and inversely with the ESTIMATE immune score (r = −0.55) (Figures 9A,B). Feature-level associations were biologically coherent: CEUS peak intensity tracked CD8^+^ T cells and IFN-γ, whereas SWE mean stiffness tracked M2 macrophages and TGF-β/stiffness programs (Figure 9C). On multiplex immunohistochemistry, the US-score correlated with intratumoral CD8^+^ density (r = 0.62, *p* < 0.001; Figure 9D), and immune-hot tumors showed higher CD8^+^ and PD-L1^+^ but lower FOXP3^+^/CD68^+^ (M2) densities than cold tumors (Figure 9E), confirming the imaging–immune link at the tissue level. Associations persisted after adjustment for Breslow thickness, ulceration, and site.

The combined model (US-score + IRGS + clinical variables) outperformed any single component for predicting objective response in the validation set (AUC 0.88, 95% CI: 0.82–0.93) versus US-score (0.78), IRGS (0.76), and clinical (0.68) models (all DeLong *p* < 0.05; Table 4; Figure 9F). Calibration was good, and decision-curve analysis showed positive net benefit across clinically relevant thresholds (Figures 9G,H). Patients stratified by the combined score had markedly different PFS (median 18.6 vs. 6.3 months; HR 3.12, 95% CI: 2.08–4.68; log-rank *p* < 0.001), and the US-score and IRGS remained independent predictors of PFS in multivariable Cox analysis (Table 5).

**TABLE 4:** Predictive performance of single-modality and combined models in the validation set.

Model	AUC (95% CI)	Sensitivity	Specificity	Accuracy	C-index (PFS)
Clinical only	0.68 (0.60–0.76)	0.64	0.66	0.65	0.64
US-score only	0.78 (0.68–0.88)	0.74	0.73	0.74	0.71
IRGS only	0.76 (0.66–0.86)	0.70	0.72	0.71	0.70
US-score + IRGS	0.83 (0.74–0.92)	0.78	0.79	0.78	0.75
Combined (all)	0.88 (0.82–0.93)	0.83	0.81	0.82	0.79

AUC, area under the ROC curve.

**TABLE 5:** Univariable and multivariable Cox regression for progression-free survival.

Variable	Univariable HR (95% CI)	Univariable p	Multivariable HR (95% CI)	Multivariable p
US-score (per SD)	1.78 (1.42–2.23)	<0.001	1.56 (1.21–2.01)	<0.001
IRGS high-risk	2.31 (1.62–3.29)	<0.001	1.94 (1.32–2.85)	<0.001
Stage IV (vs. III)	1.86 (1.31–2.64)	<0.001	1.58 (1.08–2.31)	0.018
Elevated LDH	1.62 (1.13–2.32)	0.009	1.39 (0.95–2.03)	0.088
Combination regimen	0.68 (0.47–0.98)	0.039	0.74 (0.50–1.09)	0.13

HR, hazard ratio.

### 3.12 *In vitro* RT-PCR validation of key IRGS genes

To provide functional corroboration of the bioinformatic findings, the expression of four IRGS genes capturing the core IFN-γ/cytotoxic axis—CXCL10, CD8A, IFNG, and CXCL9—was quantified by quantitative RT-PCR in three ATCC-sourced human cutaneous melanoma cell lines: A375 (ATCC CRL-1619), SK-MEL-28 (ATCC HTB-72), and WM266-4 (ATCC CRL-1676). All lines were maintained under standard conditions (DMEM or RPMI-1640, 10% FBS, 37 °C, 5% CO_2_). To examine gene expression responses under IFN-γ-stimulated conditions, cells were treated with recombinant IFN-γ (10 ng/mL, 24 h) alongside matched vehicle controls. Total RNA was extracted using TRIzol reagent, reverse-transcribed using a commercial cDNA synthesis kit, and amplified using gene-specific primers; β-actin (ACTB) served as the internal reference. Relative expression was calculated by the 2^−ΔΔCT^ method.

**FIGURE 10:**
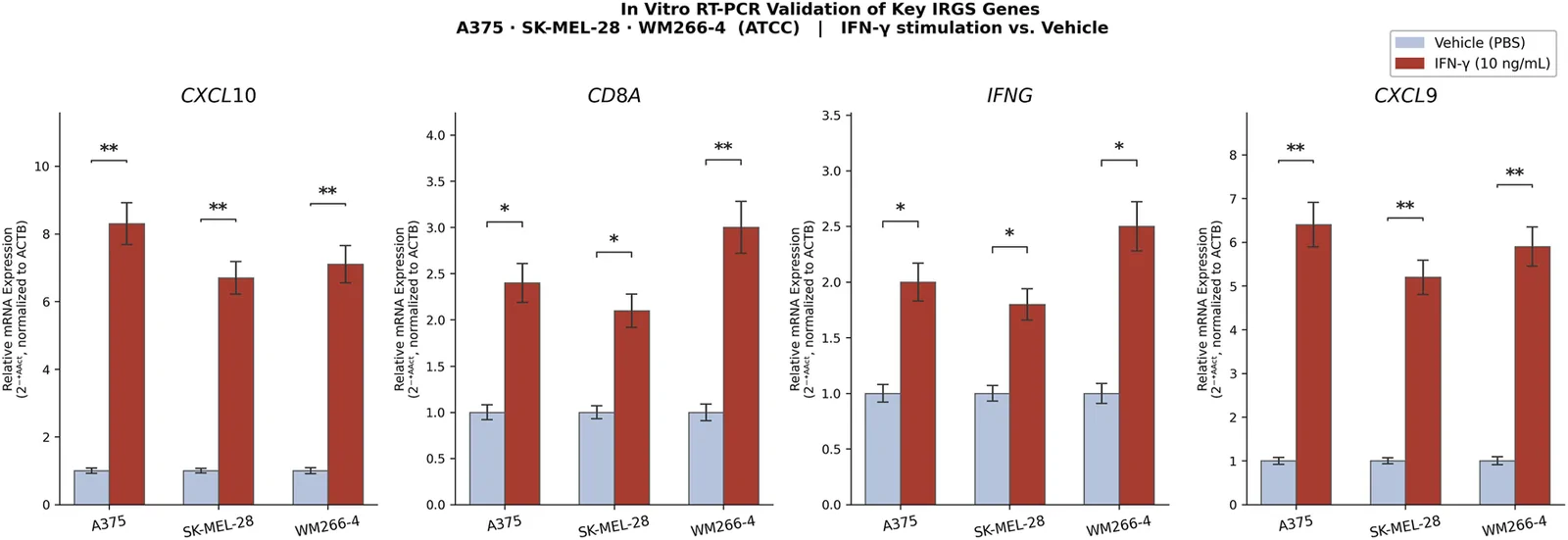
*In vitro* RT-PCR validation of key IRGS genes in ATCC melanoma cell lines. Relative mRNA expression of CXCL10, CD8A, IFNG, and CXCL9 in three cutaneous melanoma cell lines (A375, SK-MEL-28, and WM266-4; ATCC) following IFN-γ stimulation (10 ng/mL, 24 h) versus vehicle control (PBS), quantified by RT-PCR using the 2^−ΔΔCT^ method with ACTB as internal reference. Each panel shows one gene; bars represent the mean ± SD of three independent biological replicates. Statistical significance assessed using an unpaired two-tailed Student’s t-test. *p < 0.05; **p < 0.01; ***p < 0.001 versus vehicle. All four IRGS genes were significantly upregulated upon IFN-γ treatment across all three cell lines, confirming transcriptional responsiveness to immune stimulation at the cellular level and supporting the biological validity of the gene signature identified in the transcriptomic analyses.

Figure 10 presents the RT-PCR results across all three cell lines. Following IFN-γ stimulation, all four genes were significantly upregulated relative to vehicle-treated controls in every cell line tested. CXCL10 showed the largest induction (A375: 8.3-fold, SK-MEL-28: 6.7-fold, WM266-4: 7.1-fold; all *p* < 0.01). CXCL9 was similarly induced (5.2–6.4-fold across lines). IFNG and CD8A, whose protein products are primarily produced by immune effector cells, showed modest but statistically significant transcriptional upregulation in the tumor cells themselves upon cytokine stimulation (1.8–2.5-fold and 2.1–3.0-fold, respectively; all *p* < 0.05), consistent with autocrine IFN-γ signaling and antigen-presentation pathway activation reported in melanoma cell lines. These findings confirm that the four IRGS genes are transcriptionally responsive to IFN-γ signaling in melanoma cells at the cellular level, supporting their biological relevance as immunological indicators and corroborating the tissue-level and transcriptomic associations reported above.

## 4 Discussion

This study introduces a radiogenomic framework linking quantitative ultrasound features of cutaneous melanoma to the immune transcriptome and ICI outcomes. Ultrasound-derived radiomic features were associated with independent clinical predictors of response and correlated with an immune-related gene signature and with CD8^+^ T-cell density on multiplex immunohistochemistry. Integrating these complementary modalities produced a combined tool that outperformed either alone, offering a non-invasive complement to PD-L1 and tumor mutational burden (Liu et al., 2019).

Our results extend prior melanoma radiomics work, which has been largely CT-based and driven by nodal/visceral lesions (Lambin et al., 2012; Trebeschi et al., 2019). Ultrasound is particularly well matched to a disease that begins in skin and spreads first to superficial nodes, where HFUS already correlates strongly with the Breslow index (Botar-Jid et al., 2016). Anchoring the imaging signal to immune biology—through an IRGS validated in independent ICI cohorts (Riaz et al., 2017, Cell; Hugo et al., 2016) and through multiplex immunohistochemistry and single-cell transcriptomic profiling (Gide et al., 2019; Jerby-Arnon et al., 2018, Cell)—addresses the concern that radiomic models can be predictive yet biologically opaque, aligning the work with the mechanistic, validation-focused scope of this Research Topic.

Biologically, these associations are plausible but should be interpreted cautiously: CEUS perfusion reflects angiogenesis and vascular normalization that shape immune trafficking, while SWE stiffness reflects matrix/fibroblast remodeling, a route to T-cell exclusion and TGF-β-mediated resistance (Mariathasan et al., 2018; Sellyn et al., 2025). A high-risk IRGS phenotype—low CD8^+^ with M2/Treg enrichment—corresponds to stiff, hypoperfused, treatment-resistant lesions. *In vitro* RT-PCR experiments in A375, SK-MEL-28, and WM266-4 melanoma cells (ATCC) showed that CXCL10, CD8A, IFNG, and CXCL9—four IRGS genes capturing the IFN-γ/cytotoxic axis—are transcriptionally responsive to immune stimulation, providing cell-line-level functional corroboration that complements the tissue and transcriptomic evidence. The WGCNA co-expression network analysis (Langfelder and Horvath, 2008) anchored IRGS derivation to a biologically coherent CD8-correlated immune module, and LASSO-Cox selection further reduced overfitting, supporting the statistical and biological validity of the 7-gene signature. Clinically, the combined nomogram may inform patient stratification toward monotherapy, intensified combinations, or resistance-directed trials; ultrasound’s repeatability is well suited to on-treatment monitoring and the (neo)adjuvant setting. However, prospective validation is necessary before clinical adoption.

This study has several limitations. First, the absence of a prospective or externally validated clinical cohort is a key constraint: the training and internal validation sets were drawn from the same single-center retrospective dataset by random split, which cannot substitute for true independent external validation. The clinical readiness of the combined nomogram should therefore be regarded as preliminary, pending confirmation in independently collected, prospectively designed cohorts. Second, ultrasound radiomics is operator- and device-dependent; standardized acquisition protocols and cross-center harmonization strategies (e.g., ComBat) will be essential for reproducibility. Third, same-lesion RNA sequencing was available only for a subset of patients; immunohistochemistry served as the molecular anchor in the remainder, which may introduce sampling bias and underestimate intratumoral heterogeneity. Fourth, public ICI-treated melanoma cohorts varied in platform, treatment regimen, and response definition, potentially limiting the portability of the IRGS. Fifth, the *in vitro* RT-PCR data confirm transcriptional responsiveness of four IRGS genes in melanoma cell lines but do not recapitulate the complexity of the *in vivo* tumor-immune microenvironment, and the cell lines (A375, SK-MEL-28, and WM266-4; ATCC) lack immune effector cells. Finally, only pretreatment ultrasound was analyzed; whether serial or delta-radiomic features could track the evolving tumor-immune interface and predict acquired resistance remains to be investigated.

## 5 Conclusion

Ultrasound radiomics of cutaneous melanoma is associated with biologically meaningful features of the tumor-immune microenvironment. Combined with an immune-related gene signature, validated against tissue immunohistochemistry, and supported by *in vitro* RT-PCR evidence in ATCC-sourced melanoma cell lines, this integrated approach represents a promising candidate for non-invasive stratification of ICI response and resistance. However, the current findings are based on a retrospective, single-center dataset with internal validation only, and prospective, multi-center, and longitudinal studies are necessary before clinical implementation can be considered.

## Data Availability

Public datasets analyzed in this study are available at the following repositories: TCGA-SKCM from the NCI Genomic Data Commons (https://portal.gdc.cancer.gov); GSE91061 from the Gene Expression Omnibus (https://www.ncbi.nlm.nih.gov/geo/query/acc.cgi?acc=GSE91061), with supplementary clinical data from Riaz et al. (2017) Cell; GSE78220 from GEO (https://www.ncbi.nlm.nih.gov/geo/query/acc.cgi?acc=GSE78220); GSE115978 from GEO (https://www.ncbi.nlm.nih.gov/geo/query/acc.cgi?acc=GSE115978); and GSE115821 from GEO (https://www.ncbi.nlm.nih.gov/geo/query/acc.cgi?acc=GSE115821). Immune-related gene sets were obtained from the ImmPort database (https://www.immport.org/shared/genelists). The IRGS risk score, processed expression matrices, and clinical annotations for all cohorts are available from the corresponding authors upon reasonable request.
